# Changes in malaria vector bionomics and transmission patterns in the equatorial forest region of Cameroon between 2000 and 2017

**DOI:** 10.1186/s13071-018-3049-4

**Published:** 2018-08-13

**Authors:** Roland Bamou, Lili Ranaise Mbakop, Edmond Kopya, Cyrille Ndo, Parfait Awono-Ambene, Timoleon Tchuinkam, Martin Kibet Rono, Joseph Mwangangi, Christophe Antonio-Nkondjio

**Affiliations:** 1 Vector Borne Infectious Disease Unit of the Laboratory of Applied Biology and Ecology (VBID-LABEA), Department of Animal Biology, Faculty of Science of the University of Dschang, P. O. Box 067, Dschang, Cameroon; 2 Laboratoire de Recherche sur le Paludisme, Organisation de Coordination pour la lutte contre les Endémies en Afrique Centrale (OCEAC), B. P.288, Yaoundé, Cameroon; 3 Faculty of Sciences, University of Yaoundé I, P.O. Box 337, Yaoundé, Cameroon; 4 Department of Biological Sciences, Faculty of Medicine and Pharmaceutical Sciences, University of Douala, P.O. Box 24157, Douala, Cameroon; 5 Vector Biology Liverpool School of Tropical Medicine, Pembroke Place, Liverpool, L3 5QA UK; 6 KEMRI-Wellcome Trust Research Programme, Centre for Geographic Medicine Research Coast, Kilifi, Kenya; 7 KEMRI-Centre for Geographic Medicine Research Kilifi, Kilifi, Kenya; 8 Pwani University Health and Research Institute, Pwani University, Kilifi, Kenya

**Keywords:** Malaria, Transmission, Equatorial forest region, Bionomic, Cameroon, LLINs, *Anopheles*

## Abstract

**Background:**

Increased use of long-lasting insecticidal nets (LLINs) over the last decade has considerably improved the control of malaria in sub-Saharan Africa. However, there is still a paucity of data on the influence of LLIN use and other factors on mosquito bionomics in different epidemiological foci. The objective of this study was to provide updated data on the evolution of vector bionomics and malaria transmission patterns in the equatorial forest region of Cameroon over the period 2000–2017, during which LLIN coverage has increased substantially.

**Methods:**

The study was conducted in Olama and Nyabessan, two villages situated in the equatorial forest region. Mosquito collections from 2016–2017 were compared to those of 2000–2001. Mosquitoes were sampled using both human landing catches and indoor sprays, and were identified using morphological taxonomic keys. Specimens belonging to the *An. gambiae* complex were further identified using molecular tools. Insecticide resistance bioassays were undertaken on *An. gambiae* to assess the susceptibility levels to both permethrin and deltamethrin. Mosquitoes were screened for *Plasmodium falciparum* infection and blood-feeding preference using the ELISA technique. Parasitological surveys in the population were conducted to determine the prevalence of *Plasmodium* infection using rapid diagnostic tests.

**Results:**

A change in the species composition of sampled mosquitoes was recorded between the 2000–2001 collections and those of 2016–2017. A drop in the density of the local primary vectors *An. nili* and *An. moucheti* in the forest region was recorded, whereas there was an increase in the density of *An. gambiae* (*s.l*.), *An. marshallii*, *An. ziemannii* and *An. paludis*. A change in the biting behaviour from indoor to outdoor was recorded in Olama. Very few indoor resting mosquitoes were collected. A change in the night biting cycle was recorded with mosquitoes displaying a shift from night biting to late evening/early in the night. Several mosquitoes were found positive for *Plasmodium* infection, thus sustaining continuous transmission of malaria in both sites. Reduction of malaria transmission in Nyabessan was lower than that seen in Olama and associated with deforestation and the construction of a dam that may have enabled a more efficient vector, *An. gambiae* (*s.l*.), to invade the area. A high level of resistance to pyrethroids (permethrin and deltamethrin) was detected for *An. gambiae* in both sites*.* High parasite prevalence was recorded in both sites, with children of 0–16 years being the most affected. In both Olama and Nyabessan, bed net usage appeared to correlate to protection against malaria infection.

**Conclusions:**

The study shows important changes in the bionomics of vector populations and malaria transmission patterns in the equatorial forest region. The changes call for more concerted efforts to address challenges such as insecticide resistance, environmental modifications or behavioural changes affecting the performance of current control measures.

## Background

Malaria still has a devastating impact on public health and welfare on the African continent. In Cameroon, over 30% of the population suffer from yearly malaria attacks resulting in 4000 to 10,000 deaths annually [1]. Long-lasting insecticidal nets (LLINs) are the main tools used across the country for malaria vector control. Over the last decade, up to three important mass distribution campaigns have been conducted [2]. The first, conducted in 2004, saw the free distribution of up to two million nets to children under five years and pregnant women, whereas insecticide treated nets (ITNs) were subsidised for the other age groups [3]. The second campaign conducted in 2011 included the free distribution of over 8 million LLINs to the whole population, and the third campaign conducted in 2015 included free distribution of over 12 million nets countrywide [4]. It is estimated that > 60% of the population currently own treated nets [5, 6], and that 50–70% of the population use nets regularly [7]. Although scale-up of malaria control strategies including mass distribution of treated nets across the continent contributed over the last decade to a significant decrease in malaria morbidity and mortality [1], the effectiveness of these measures is threatened by the rapid expansion of insecticide resistance in vector populations [8–12], change in vector feeding, biting and resting behaviour and the diversity of the vectorial system. Across Africa, several studies have reported different behavioural changes in mosquitoes affecting treated nets efficacy. In Benin, Moiroux et al. [13] reported changes in the biting time of *An. funestus* from midnight to dawn after LLIN scale-up. In Tanzania and Kenya, *An. arabiensis*, the main vector in these areas, was reported to be less affected by control measures because of its high zoophagic and exophilic behaviour [14–17]. Some populations of *An. arabiensis* were reported to avoid fatal insecticide exposure by entering and rapidly exiting houses containing indoor residual spraying (IRS) and LLINs or limiting the feeding time [18–20]. Yet, it is still unknown whether behavioral changes reported so far are true genetic changes resulting from insecticide selection or the expression of pre-existing plastic behavioral traits in response to modified resource availability, also known as resilience [21] or altered taxonomic composition deriving from suppression of the most vulnerable taxa [22]. In addition to these changes, rapid expansion of insecticide resistance in mosquito populations was reported across the continent [23–26]. A recent review on the status of insecticide resistance in Cameroon indicated that apart from organophosphates, most compounds used in public health are largely affected by insecticide resistance [2]. In addition to target site insensitivity being highly prevalent in *An. gambiae*, several sets of insecticide detoxification genes have been identified in *An. gambiae*, *An. arabiensis* and *An. funestus* [8, 26]. Another important factor which could affect the performance of vector control tools and has not been scrupulously evaluated is the diversity of the vectorial system. In Cameroon, up to 15 species are permanent or occasional malaria vectors [27]. In the northern part of the country situated in the dry savannah and sahelian region, malaria transmission is seasonal and vectored by species such as *An. arabiensis*, *An. gambiae* and *An. funestus* as the main vectors [28, 29]. Other species such as *An. rufipes* or *An. pharoensis* also can be involved in disease transmission [30–32], while in the southern part of the country situated in the forest region, malaria transmission is perennial [5] with a high diversity of species responsible for transmission. In addition to the dominant vectors within this area (*An. gambiae*, *An. coluzzii*, *An. funestus*, *An. moucheti* and *An. nili*) several secondary vectors such as *An. ovengensis*, *An. paludis*, *An. ziemanni* and *An. marshallii* contribute either seasonally or occasionally to malaria transmission [27, 33].

In the forested regions of Cameroon, it is estimated that over 80% of households own at least one net [34]. Over a number of years, the area has been affected by increased deforestation following extension of population settlements, construction of roads or dams, and changing agricultural practices with the cutting down of trees, yet the influence of these changes on the vectorial system dynamics and malaria transmission patterns have not been fully examined. The present study was conducted to assess the evolution of mosquito bionomics and malaria transmission patterns in association with the changing use of LLINs and other related changes in the area by comparing samplings undertaken in 2000–2001 and 2016–2017, before and after large scale campaigns of treated net distribution to communities.

## Methods

### Study sites

**Fig. 1:**
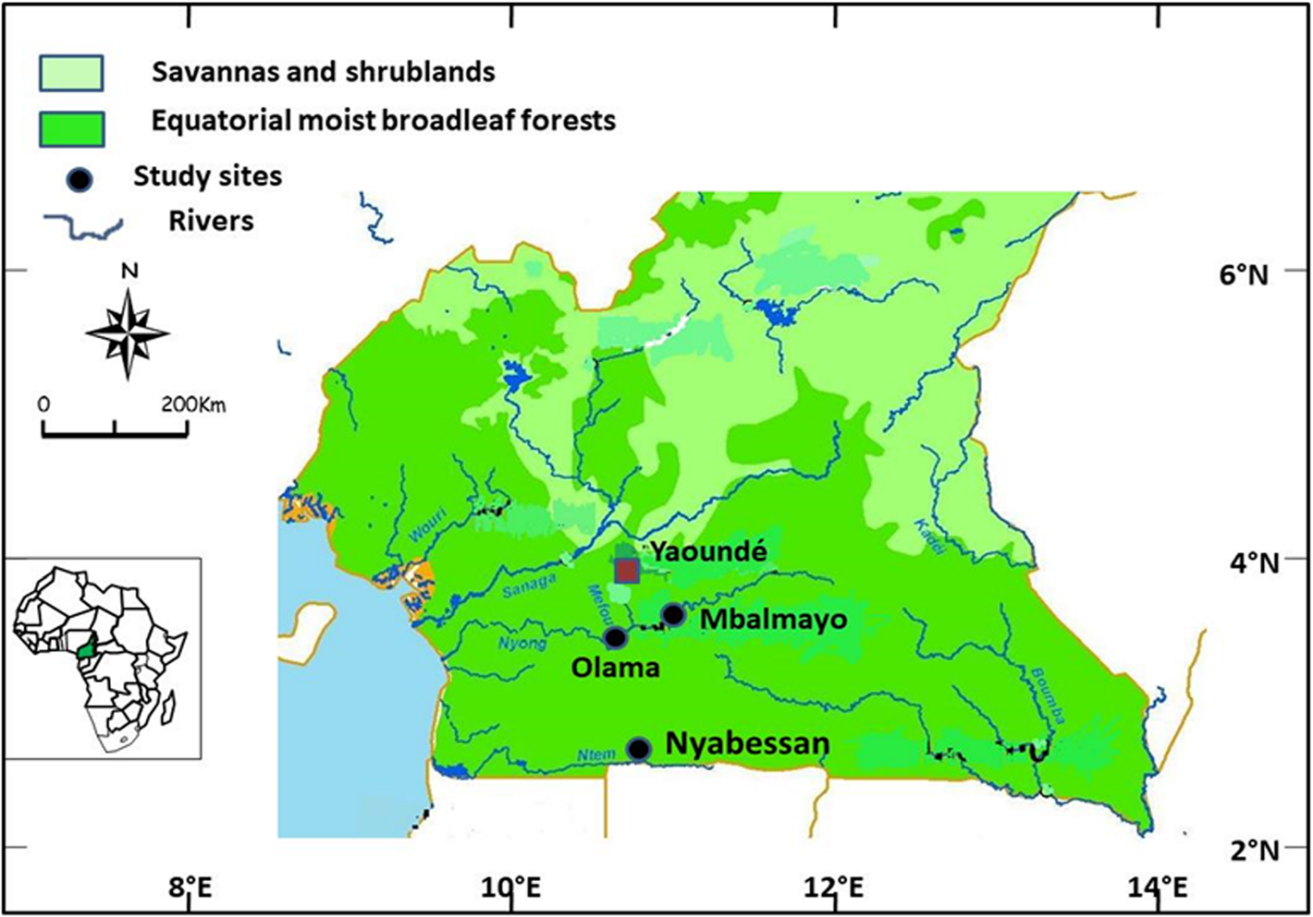
Map showing study sites

The study was conducted within the villages of Olama and Nyabessan in the equatorial forest region of Cameroon (Fig. 1). Olama village (3°24'N, 11°18'E), is situated 65 km south of Yaoundé on the Nyong River. Nyabessan (2°80'N, 10°25'E) is situated 220 km south of Yaoundé on the Ntem River. Houses in both villages are mainly constructed with mud walls and roofs of corrugated iron or planks. Houses have large eaves leaving sufficient space for mosquitoes to fly in. Nyabessan is characterized by the presence of *An. gambiae*, *An. nili* and *An. moucheti* as the dominant malaria vectors [33], whereas in Olama, the main vector is *An. moucheti* with *An. gambiae* playing a minor role [35]. Nyabessan and Olama display high and perennial malaria transmission patterns. Both sites are located within the Congo-Guinean phytogeographic zone, characterized by a typical equatorial climate with two rainy seasons extending from March to June and September to November. Mean annual rainfall ranges between 1600–1800 mm. Although both villages experience deforestation at a limited scale, recent construction of a dam within Nyabessan from 2012 to 2016 has considerably changed its landscape with much reduction in vegetation cover compared to earlier times.

### Hypothesis

We hypothesized that changes which occurred in the forest region between 2000 and 2017, including the free distribution campaigns of treated nets to the population (conducted in 2004, 2011 and 2015), environmental changes (deforestation, construction of a dam and roads, extension of population settlements, etc.) and human behavioural factors, may have deeply affected mosquito population bionomic and malaria transmission patterns in the equatorial forest region. To test this hypothesis we studied a range of outcome measures (mosquito species distribution, species diversity, mosquito densities, mosquito biting and resting behaviour, infection and transmission rate) and compared these between the collections of 2000–2001 and those of 2016–2017.

### Adult mosquito collections and field processing

Mosquito collections were conducted using two sampling methods in both Olama and Nyabessan; these included human landing catches (HLC) carried out from 19:00 to 06:00 h indoors and outdoors, and indoor pyrethrum spray collection (PSC). In 2000–2001, human landing collections were performed in 3 randomly selected sites (households) on two consecutive nights once every two months, whereas in 2016–2017, mosquitoes were collected in 4–10 households per village for three consecutive days once every two or three months. Mosquitoes collected were placed into separate bags, labelled according to the site, night and hour of collection. The bags were kept in a cooler box for preservation while in the field. Indoor spray collections were conducted early in the morning (06:00 to 10:00 h) in 5 to 10 houses per village per day.

After collection, mosquitoes were sorted into genus and species using the morphological identification keys of Gillies & Coetzee [36] and Gillies & De Meillon [37]. Specimens were stored individually in labeled tubes containing desiccant, archived at -20 °C until ready for processing in the laboratory at Yaoundé.

### Laboratory processing of mosquitoes

Members of the *An. gambiae* complex were identified using the molecular diagnostic tools previously described [38]. DNA was extracted from a mosquito leg and/or wing and used for analysis. Enzyme linked immunosorbent assays (ELISA) were used to determine the presence of *Plasmodium* parasites in the head and thorax of anopheline mosquitoes [39–42]. Blood-meal source identification in fed females from pyrethrum spray catches was done by ELISA for differentiating blood from either human, bovine, ovine (sheep and goat), equine (horse and donkey), pig or chicken hosts [43].

### Larval collections

Larval collections were undertaken in different habitats including temporary water collections, puddles and semi-permanent sites, to avoid oversampling single mosquito families. Mosquitoes collected from these different breeding sites were pooled and reared together. Adult females were used for susceptibility bioassays 2 to 4 days after emerging from larvae.

### Assessment of mosquito susceptibility to pyrethroids

Evaluation of mosquito susceptibility to insecticides was undertaken following WHO guidelines [44]. Deltamethrin and permethrin, the two compounds used for impregnating bednets in Cameroon, were used for the analysis. *Anopheles gambiae* females aged 2–4 days, reared from larvae collected in the field, were kept in batches of 20–25 mosquitoes per tube and exposed to insecticide-impregnated papers for 1 h. The insecticide-susceptible *An. gambiae* Kisumu strain was used as a control to measure the effectiveness of the impregnated papers. The numbers of mosquitoes knocked down by the insecticide were recorded every 10 min during exposure. After 1 h of exposure, mosquitoes were fed with a 10% glucose solution and the number of dead mosquitoes was recorded 24 h post-exposure. Mosquitoes subjected to untreated papers were systematically run as controls. The mortality rates were corrected using Abbot’s formula [45] whenever the mortality rate in the controls ranged between 5–20%. WHO criteria [44] were used to evaluate the resistance and susceptibility status of the test mosquito population. Three classes of insecticide susceptibility were defined: insecticide resistant (< 90%); insecticide tolerant (90–97%); and insecticide susceptible (> 97%).

Susceptibility tests conducted in Mbalmayo in 2000 were also undertaken with females aged 2–4 days (see Etang et al. [46] for further details).

### Parasitological analysis

Malaria rapid diagnostic tests (mRDTs) were conducted in households who consented to the study to determine malaria prevalence in the population within the two villages. Information concerning the age, sex, bednet usage and occupation were recorded from each consented participant during the survey. Malaria parasite screening in blood samples was performed using the SD Bioline Malaria Ag. *Plasmodium falciparum* rapid diagnostic test kit which has 98% specificity and 99.5% sensitivity [47].

### Entomological indicators and data analysis

The human biting rate (HBR) for mosquitoes was estimated as the number of mosquitoes collected per man per night. *Plasmodium* infection rates in *Anopheles* were calculated as female *Anopheles* found infected by *Plasmodium falciparum* circum-sporozoite protein (CSP) antigens over screened mosquitoes. The annual entomological inoculation rate (EIR) was calculated by multiplying the human biting rate by the circum-sporozoite rate and by the number of days of the year.

Statistical analysis for the comparison of population mean, significance levels, odds ratio, Chi-square tests and confidence interval estimation were performed using MedCalc v.14.8.1 software.

To assess the level of reduction of the density of host-seeking mosquitoes, a generalized linear mixed model (GLMM) fitting a negative binomial distribution was applied. For these analyses, the density of mosquitoes collected per man per night was considered as the response variable. Fixed variables were the year and site of collection (indoor/outdoor). The month of collection was considered as a random intercept to adjust for sampling variations across years. The analysis was conducted using the *lme4* package of the software R version 3.4.0 [48].

## Results

### Mosquito distribution

#### Composition of the mosquito fauna in study sites

For the 2000–2001 collections, a total of 2507 anophelines were collected in Nyabessan using 72 man-night collectors and 4207 anophelines collected in Olama using 108 man-night collectors. For the 2016–2017 collections, 10,180 anophelines in Nyabessan and 5057 in Olama were collected using 258 man-night collectors at each site. The density of mosquitoes in Nyabessan was 34.82 ± 0.97 bites/man/night in the 2000–2001 collection and 39.45 ± 0.56 bites/man/night in the collection of 2016–2017, although the difference was not significant (Generalized linear mixed model, Wald *χ*^2^ = 0.18, *P* = 0.66). In Olama, a significant decrease in mosquito density was recorded with densities decreasing from 38.95 ± 0.46 bites/man/night in the collections of 2000–2001 to 19.59 ± 0.48 bites/man/night (Wald *χ*^2^ = 16.27, *P* < 0.0001). Analysis of mosquito species composition displayed important variation between the two sampling periods (Table 1). Species displaying marked density variations between catches included *An. paludis* and *An. gambiae* in Nyabessan while *An. mashallii*, *An. paludis* and *An. ziemannii* varied in Olama. In Nyabessan, the most prevalent species recorded were *An*. *gambiae*, *An. moucheti*, *An. paludis* and *An. nili*. In Olama the most prevalent species were *An. moucheti*, *An. marshallii*, *An. paludis* and *An. ziemannii*.

**Table 1:** Mosquito composition in 2000–2001 and 2016–2017 in Olama and Nyabessan, calculated from human landing catches

	2000–2001		2016–2017	
	Indoor	Outdoor	Indoor	Outdoor
Nyabessan^a^				
*An. gambiae*	42	61	1327	1635
*An. moucheti*	149	712	1474	1885
*An. marshallii*	2	91	25	34
*An. paludis*	0	3	869	1425
*An. ziemannii*	-	-	16	5
*An. nili*	184	1263	624	861
Total	377	2130	4335	5845
Mean^a^ (95% CI)	10.47 (9.85–11.09)	59.17 (57.21–61.13)	33.6 (32.84–34.36)	45.31 (44.48–46.14)
Olama^b^				
*An. gambiae*	13	13	20	3
*An. moucheti*	2743	1375	1293	1379
*An. marshallii*	-	9	446	426
*An. paludis*	9	32	427	426
*An. ziemannii*	3	2	337	297
*An. nili*	3	5	1	2
Total	2771	1436	2524	2533
Mean^c^ (95% CI )	51.31 (50.51–52.11)	26.59 (26.03–27.15)	19.56 (18.91–20.21)	19.62 (18.92–20.32)

^a^Nyabessan: 2000–2001 (mosquitoes collected using 72 man-nights); 2016–2017 (mosquitoes collected using 258 man-nights)

^b^Olama: 2000–2001 (mosquitoes collected using 108 man-nights); 2016–2017 (mosquitoes collected using 258 man-nights)

^c^Average bites per human per night

#### Molecular identification of sibling species

Molecular identification of sibling species within the *An. gambiae* complex using SINE PCR indicated the presence of both *An. gambiae* and *An. coluzzii* in the two sites, whereas in previous collections, *An. coluzzii* was rare in Nyabessan (Table 2). In the 2016–2017 collection, little seasonal variation in the distribution of *An. coluzzii* and *An. gambiae* were recorded in Nyabessan.

**Table 2:** Distribution of members of the *An. gambiae* complex collected in 2000–2001 and 2016–2017 in Olama and Nyabessan

	Nyabessan				Olama			
	2000–2001		2016–2017		2000–2001		2016–2017	
	*n*	%	*n*	%	*n*	%	*n*	%
*An. gambiae*	29	100	184	54.5	6	40	6	75
*An. coluzzii*	0	0	154	45.5	9	60	2	25
Total	29	100	338	100	15	100	8	100

#### Seasonal variation of mosquito densities

**Fig. 2:**
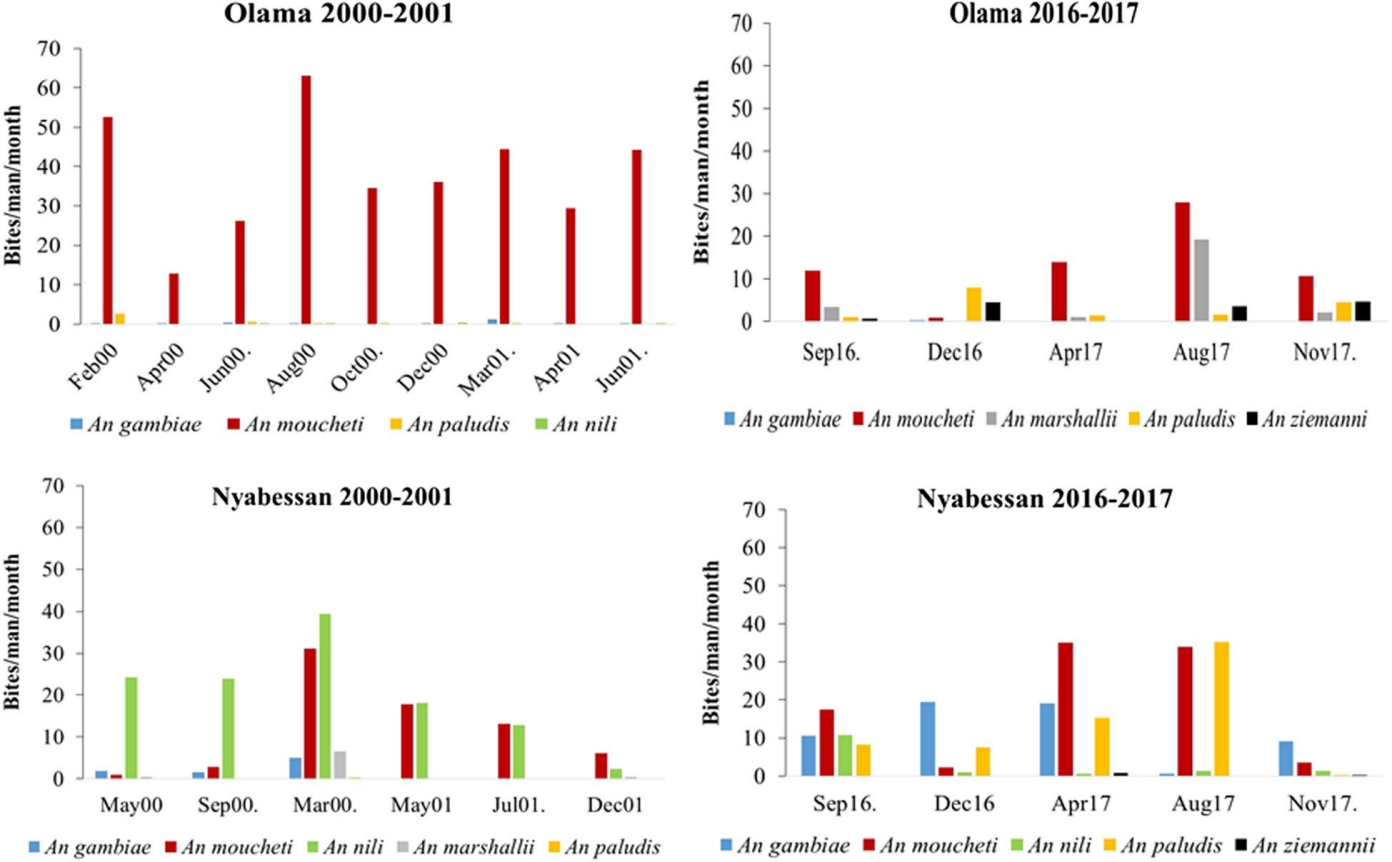
Seasonal variation of anopheline species densities in Olama and Nyabessan in 2000–2001 and 2016–2017

In the 2000–2001 collection (before the introduction of bednets), species such as *An. moucheti* in Olama and *An. moucheti* and *An. nili* in Nyabessan were always predominant across all seasons. However, during the subsequent sampling of 2016–2017, this dynamic was no longer recorded, with significant changes in species composition from one season to the other in both sites (Fig. 2).

### Mosquito biting behaviour

#### Indoor-outdoor biting behaviour

**Fig. 3:**
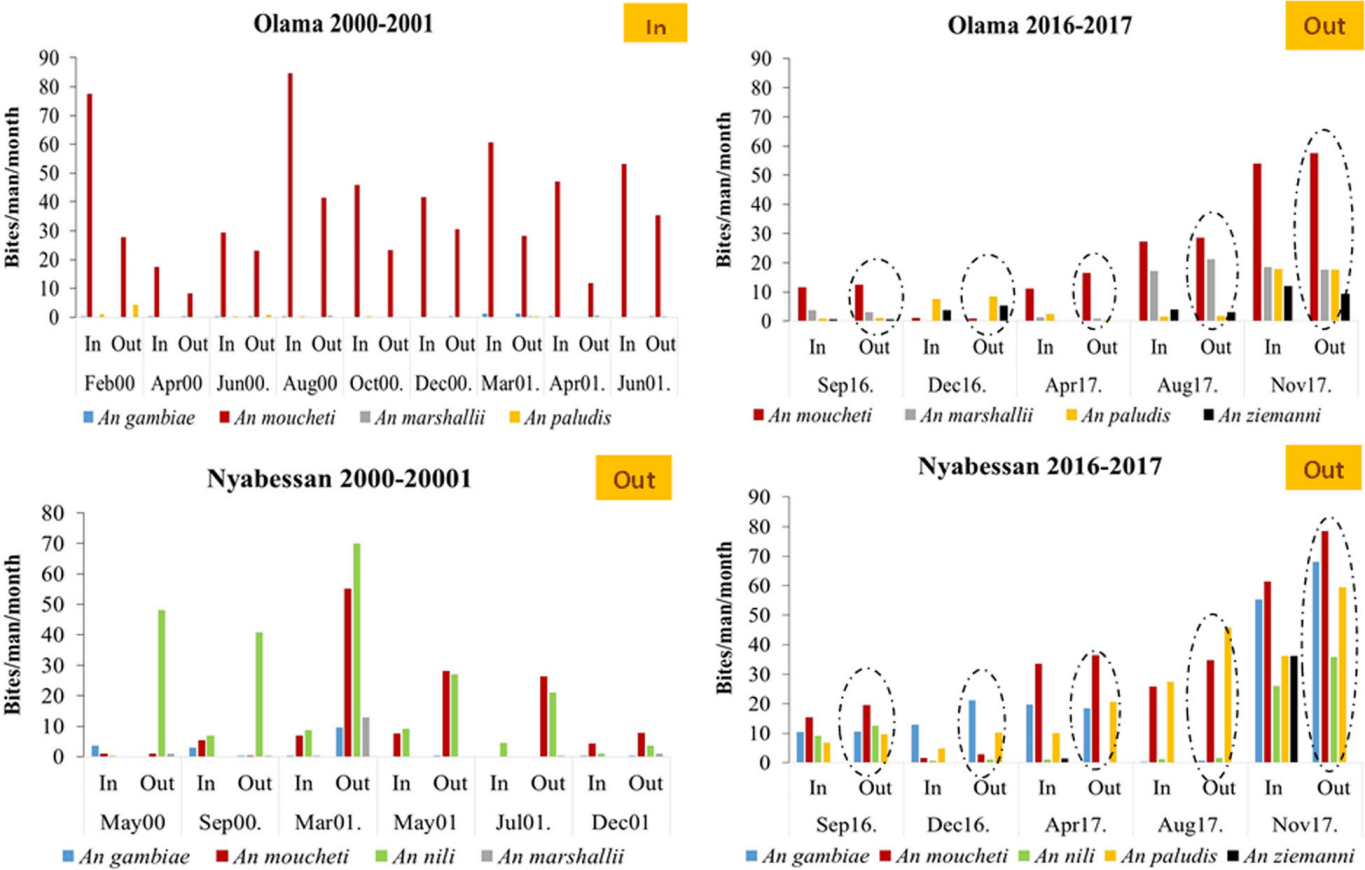
Comparison of mosquito indoor (In) and outdoor (Out) biting behaviour in Olama and Nyabessan between collections of 2000–2001 and those of 2016–2017

In the 2000–2001 survey, a high prevalence of indoor biting anophelines was recorded in Olama whereas on the contrary, anopheline preference was for outdoor biting in Nyabessan. In the 2016 to 2017 collection, a change in the biting behaviour was recorded in Olama with more mosquitoes detected feeding outdoors. In Nyabessan, mosquitoes were still found feeding outdoors but the proportion of anophelines feeding outdoors was lower in the 2016–2017 collections (57.39%) compared to the collections of 2000–2001 (84.96%) (Fig. 3).

#### Mosquito night-biting cycle

**Fig. 4:**
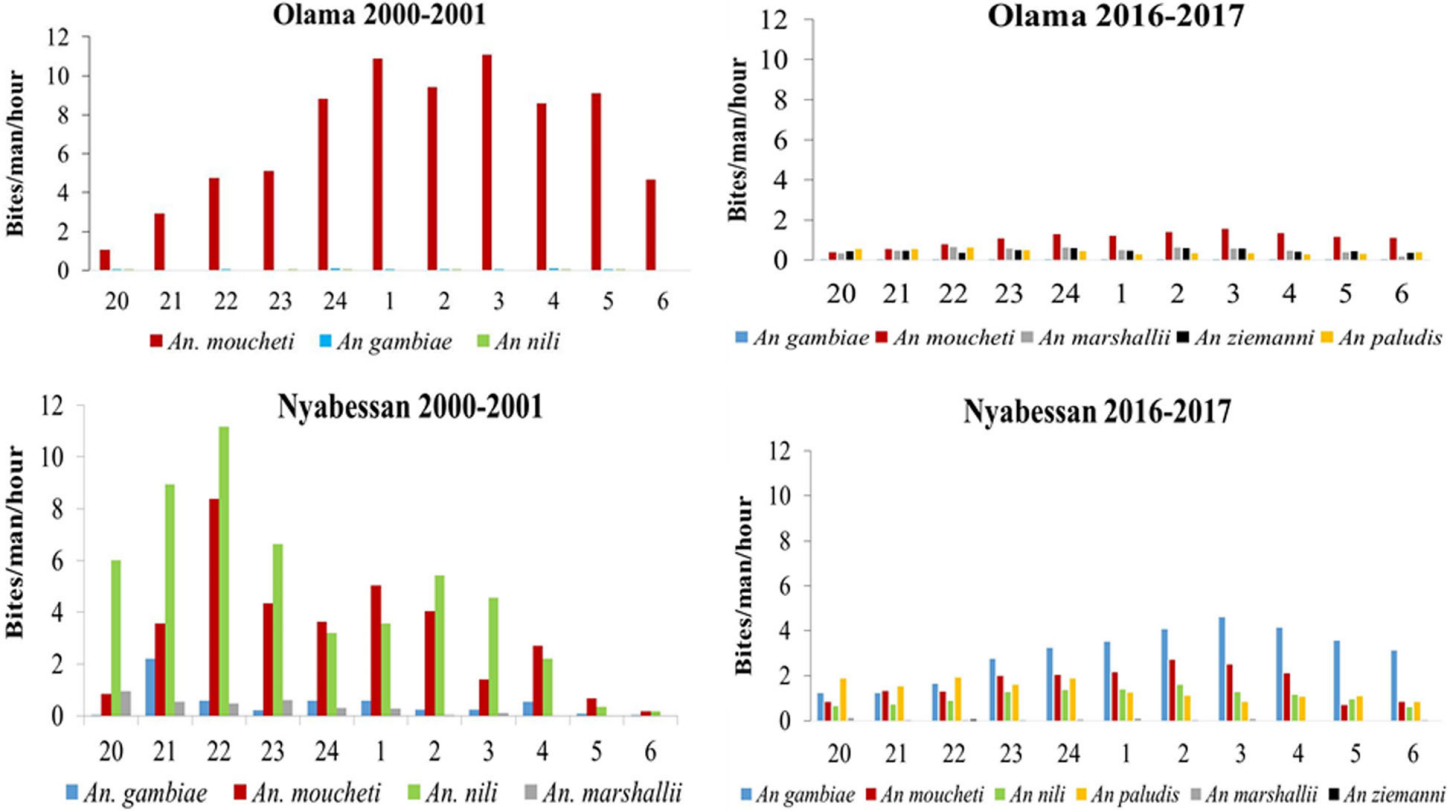
Night-biting cycle of anophelines collected in Olama and Nyabessan in 2000–2001 and 2016–2017

From the 2000–2001 collection it appeared that in Olama, most anopheline bites occurred during the second part of the night (from 24:00 to 6:00 h). In Nyabessan, high biting rates were instead recorded during the first part of the night. This change in night-biting patterns was associated with a decrease in main vector densities in the collections of 2016 to 2017. In Olama, the main species, *An. moucheti*, was found to bite both during the first and second part of the night, with a different biting pattern recorded for *An. paludis*, *An. ziemannii* and *An. marshallii* which were found to bite mostly in the first part of the night. In Nyabessan, an increased biting rate in the second part of the night was recorded for *An. gambiae* and *An. moucheti* whereas other species were biting predominantly during the first part of the night (Fig. 4).

### Pyrethrum spray collections

In the collection of 2000–2001, 444 anophelines were collected in Olama after spraying 37 rooms, and 244 were collected in Nyabessan after spraying 26 rooms: an average of 12 anophelines collected per room in Olama and 9.38 anophelines per room in Nyabessan (Table 3). *Anopheles moucheti* predominated from collections from both Olama and Nyabessan. In 2016–2017 a decrease in the densities of indoor resting anophelines was seen with only 14 collected after spraying 80 rooms in Olama and 132 anopheline collected after spraying 85 rooms in Nyabessan making an average of 0.175 anophelines per room in Olama and 1.54 anophelines per room in Nyabessan. Interestingly, *An. gambiae* was the main species found resting indoors; other species were scarce.

**Table 3:** Mosquitoes collected in Olama and Nyabessan using pyrethrum spray collections

Species	2000–2001		2016–2017	
	*n*	*n*/room	*n*	*n*/room
Nyabessan				
*An. gambiae*	40	1.5	126	1.480
*An. moucheti*	189	7.3	2	0.023
*An. marshallii*	2	0.1	1	0.012
*An. nili*	13	0.5	2	0.023
Total	244	9.38	131	1.540
Olama				
*An. gambiae*	12	0.32	14	0.175
*An. moucheti*	432	11.67	0	0
*An. marshallii*	0	0	0	0
*An. nili*	0	0	0	0
Total	444	12	14	0.175

*Abbreviations*: *n* number of mosquitoes collected; *n*/*room* number of mosquitoes per room

### Blood-feeding analysis

The human blood-feeding index of anophelines collected in the period 2016–2017 was compared to those collected in 2000–2001. Anophelines collected during these two periods displayed a high human blood index (Table 4). No significant difference in the human blood index was recorded between the two periods in the two sites (*P* > 11%).

**Table 4:** Human blood index (HBI) of mosquitoes collected resting indoor in Olama and Nyabessan

	2000–2001			2016–2017		
	Tested	Human	HBI (%)	Tested	Human	HBI (%)
Nyabessan						
*An. gambiae*	18	18	100	92	69	75
*An. nili*	10	10	100	2	2	100
*An. moucheti*	56	56	100	13	13	100
*An. marshallii*	2	2	100	–	–	–
Olama						
*An. gambiae*	4	4	100	8	8	100
*An. nili*	186	186	100	3	3	100
*An. moucheti*	–	–	–	12	12	100
*An. paludis*	–	–	–	2	2	100

*Abbreviation*: *HBI* proportion of blood meals on humans

### *Plasmodium* infection in mosquitoes

A total of 19,814 mosquitoes (11,051 and 8763 from Nyabessan and Olama, respectively) collected in both periods were analysed for the presence of *Plasmodium* infections, out of which 297 were found infected (1.5%). Six species were found infected including *An. gambiae*, *An. moucheti*, *An. nili*, *An. paludis*, *An. ziemannii* and *An. marshallii* (Table 5).

**Table 5:** *Plasmodium* infections in mosquitoes from Olama and Nyabessan

	2000–2001			2016–2017		
	Tested	Infected	% (95% CI)	Tested	Infected	% (95% CI)
Nyabessan						
*An. gambiae*	534	13	2.4 (1.3–4.2)	2507	124	4.9 (4.1–5.9)
*An. marshallii*	–	–	–	54	1	1.8 (0.05–10.3)
*An. moucheti*	681	14	2.1 (1.1–3.4)	2731	20	0.7 (0.4–1.1)
*An. nili*	1451	10	0.7 (0.3–1.3)	1268	12	0.9 (0.5–1.6)
*An. paludis*	–	–	–	1942	13	0.7 (0.3–1.1)
*An. ziemannii*	–	–	–	21	0	0.0 (0–17.6)
Total	2666	37	1.4 (1.0–1.9)	8523	170	2.0 (1.7–2.3)
Olama						
*An. gambiae*	37	4	10.8 (2.9–27.7)	23	0	0.0 (0–16.0)
*An. marshallii*	8	1	12.5 (0.3–69.6)	766	1	0.13 (0.0–0.7)
*An. moucheti*	4084	85	2.08 (1.7–2.6)	2418	15	0.62 (0.3–1.0)
*An. nili*	–	–	–	2	0	0.0 (0–100)
*An. paludis*	–	–	–	710	5	0.70 (0.2–1.6)
*An. ziemannii*	–	–	–	715	2	0.28 (0–1.0)
Total	4129	90	2.2 (1.7–2.8)	4634	23	0.50 (0.3–0.7)

The infection rate in Nyabessan varied from 1.4% in 2000–2001 to 2% in 2016–2017 whereas in Olama it varied from 2.2% in 2000–2001 to 0.5% in 2016–2017.

### Malaria transmission pattern

**Fig. 5:**
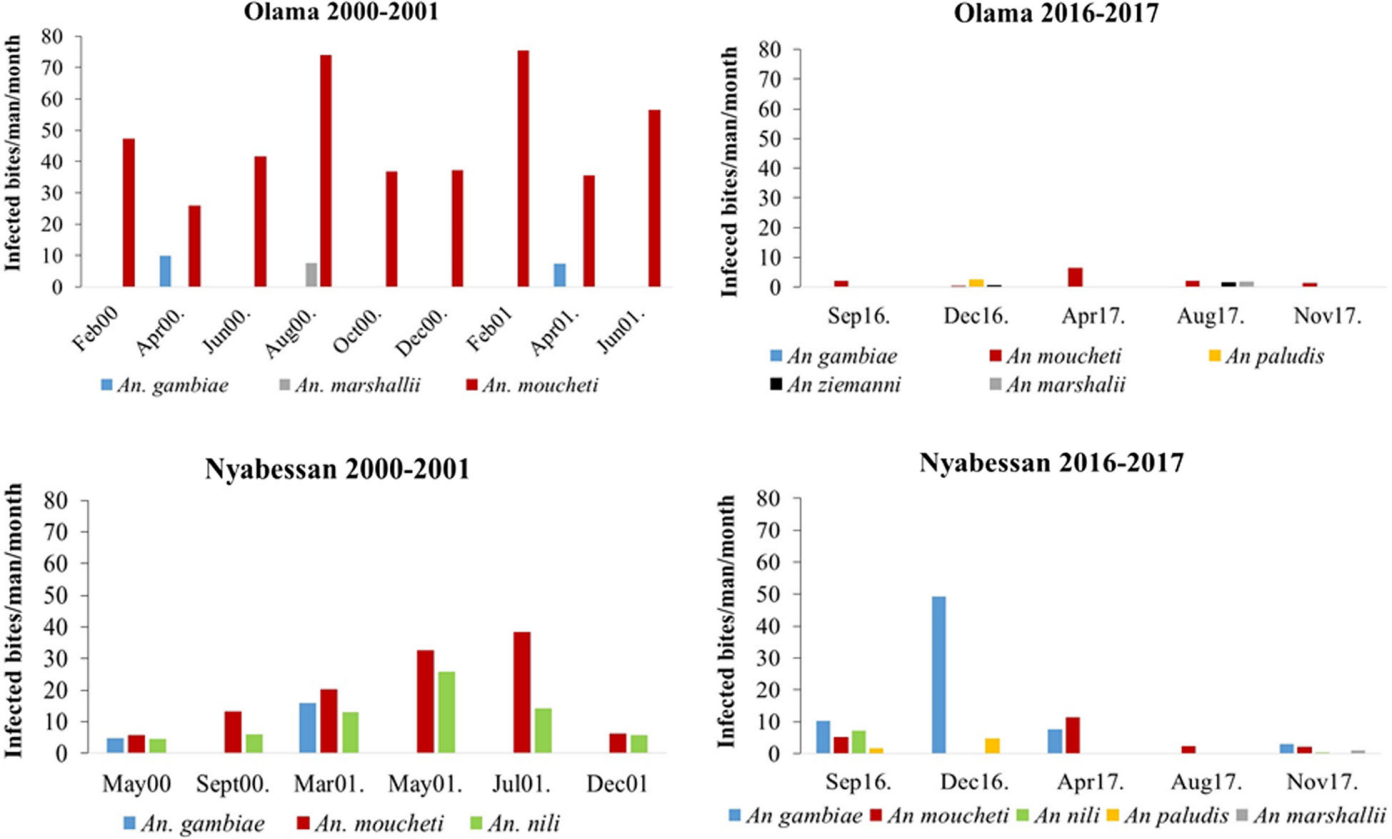
Malaria transmission pattern in Olama and Nyabessan in 2000–2001 and 2016–2017

In Nyabessan, a reduction of 26% of the entomological inoculation rate (EIR) from 0.93 infected bites/man/night in collections of 2000–2001 to 0.69 infected bites/man/night in collections of 2016–2017 was recorded. In Olama, a reduction of 92% of the EIR from 1.69 infected bites/man/night in collections of 2000–2001 to 0.12 infected bites/man/night in collections of 2016–2017 was recorded. Important variation in the dynamic of species involved in malaria transmission was also recorded in both sites (Fig. 5).

### Mosquito susceptibility to permethrin and deltamethrin

A total of 179 *An. gambiae* females aged 2–4 days in Olama and 414 in Nyabessan were exposed to 0.05% deltamethrin and 0.75% permethrin. High resistance to both permethrin and deltamethrin with mortality rates of less than 63% in both Olama and Nyabessan was detected (Table 6). Susceptibility analyses conducted in the site of Mbalmayo close to the collection sites in 2000 indicated a mortality rate of 98.8% to permethrin and 100% to deltamethrin [46]. Eighty-four *An. gambiae* females from Nyabessan were exposed to bendiocarb 0.1% and a 100% mortality rate was recorded. A sample of 332 *An. gambiae* (*s.l.*) from Nyabessan were further processed to assess the frequencies of *An. gambiae* and *An. coluzzii* in dead and surviving mosquitoes. Out of the 234 mosquitoes who survived the tests, 44.9% (105/234) were *An. coluzzii* and 55.1% (129/234) *An. gambiae*. Among the dead mosquitoes (susceptible), 48% (47/98) were *An. coluzzii* and 52% (51/98) were *An. gambiae*. No significant difference in the susceptibility level of the two species was recorded (*P* = 0.69). In Olama, the 72 specimens tested including resistant and dead were all *An. gambiae*. In the sample from Mbalmayo in 2000, out of 45 specimens processed 43 (95.6%) were *An. coluzzii* and 2 (4.4%) *An. gambiae* [46].

**Table 6:** Susceptibility level of mosquitoes to deltamethrin, permethrin and bendiocarb in Olama and Nyabessan in 2016–2017 and in Mbalmayo in 2000

Insecticide	2016–2017						2000		
	Nyabessan			Olama			Mbalmayo (village close to both sites) [46]		
	*n*	Dead	% (95% CI)	*n*	Dead	% (95% CI)	*n*	Dead	% (95% CI)
Deltamethrin 0.05%	258	131	50.8 (42.4–60.2)	109	68	62.4 (48.4–79.1)	93	93	100 (80.7–122.5)
Permethrin 0.75%	156	42	26.9 (19.4–36.4)	70	21	30 (18.6–45.9)	–	–	–
Permethrin 1%	–	–	–	–	–	–	81	80	98.8 (78.3–122.9)
Bendiocarb 0.1%	84	84	100 (79.8–123.8)	–	–	–	–	–	–

*Abbreviations*: *n*, number of mosquitoes tested; %, mortality rate; 95% CI, 95% confidence interval

### Use of LLINs and *Plasmodium* infection prevalence

Since no similar survey was undertaken before, data presented here describe results from surveys conducted during the period of 2016–2017. In total, 836 individuals were included in the study (430 in Olama and 406 in Nyabessan). Out of the 836 people tested, 351 were found infected with *P. falciparum* making an overall prevalence of 42%. High infection rates were recorded in children aged 0 to 16 years in both sites (Table 7).

**Table 7:** Distribution of *Plasmodium falciparum* infection cases according to different age groups in Olama and Nyabessan

Site	Age (years)	Tested	RDT+	RDT-	% infection (95% CI)
Olama	0–5	140	32	108	23 (15.6–32.3)
	5–10	141	55	86	39 (29.4–50.8)
	11–16	88	21	67	24 (14.8–36.5)
	> 16	61	1	60	2 (0.4–9.1)
	Total	430	109	318	25 (20.8–30.6)
Nyabessan	0–5	160	94	66	59 (47.5–71.9)
	5–10	99	73	26	74 (57.8–92.7)
	11–16	63	42	21	67 (48.1–90.1)
	> 16	84	17	67	20 (11.8–32.4)
	Total	406	242	164	60 (52.3–67.6)
Both	0–5	300	126	174	42 (52.5–75.0)
	5–10	240	128	112	53 (44.5–63.4)
	11–16	151	63	88	42 (32.1–53.4)
	> 16	145	18	127	12 (7.4–19.6)
	Total	836	351	482	42 (37.7–46.6)

*Abbreviation*: *RDT* rapid diagnostic tests

The level of infection between users and non-users of treated nets was assessed using rapid diagnostic tests. From the analysis, it appeared that the lack of bednet usage increased the risk of malaria infection 2.2-fold (Table 8).

**Table 8:** *Plasmodium* infection prevalence between users and non-users of LLINs

Sites	Use of LLINs	RDT+	RDT-	OR (95% CI)	*P*-value
Olama (*n* = 405)	Yes	84	274	1	0.06
	No	17	30	1.8 (0.97–3.51)	
Nyabessan (*n* = 380)	Yes	136	129	1	0.06
	No	71	44	1.5 (0.97–2.39)	
Both (*n* = 785)	Yes	220	403	1	<0.0001
	No	88	74	2.17 (1.53–3.09)	

*Abbreviation*: *RDT* rapid diagnostic tests

## Discussion

Changes in mosquito bionomics and malaria transmission patterns were recorded in both Olama and Nyabessan and were consistent with the possible influence of treated net use and other additional factors [1, 16, 32, 49, 50]. In Cameroon, over 60% of the population is considered to possess or to be covered by treated bednets [4, 6]. Yet, malaria transmission is highly heterogeneous across the country. It is considered to be perennial in the southern part of the country situated the equatorial forest region where rainfall can last up to eight months. In the northern part of the country where the dry savannah area is situated and less than six months of rain are registered annually, malaria transmission is seasonal. Yet, this part of the country is exposed to frequent outbreaks during the rainy season. In 2012 and 2013, rainy season outbreaks resulting in over 260,000 malaria cases were reported with 1600 deaths. Considering the high vulnerability of the area, the government also included in their arsenal of control measures, seasonal malaria chemoprevention during the rainy seasons in addition to LLIN use since 2014 [5].

Although Olama and Nyabessan are just 150 km apart in the equatorial forest region, different biting, resting and distribution patterns were recorded for the various malaria vectors inhabiting these regions which could point to the influence of both local environmental changes and LLIN use. Indeed, in Nyabessan, the construction of a water dam within the area resulted in a high influx to the local population both during and after the dam construction. Both dam construction and population movements contributed to increased deforestation of the area. An immediate consequence of this could be the creation of suitable breeding opportunities for *An. gambiae* (*s.l*.) which is now the predominant species in the area. In Olama, although the population increased over time, no drastic changes in the forest cover was recorded, thus leading to the scarcity of *An. gambiae* in the area, consistent with previous findings [51]. Different seasonal distribution patterns contrasting with previous observations were recorded. Seasonal reductions in *An. moucheti* or *An. nili* densities were compensated by increases in the densities of species such as *An. marshallii* and/or *An. paludis* which share similar breeding habitats. The following could likely suggest increased competition at the larval stage between these species or the probable influence of LLIN selection on adult *An. moucheti* and *An. nili* longevity in the natural environment. Yet, this still deserves further investigation. Important changes in the level of endo/exophagy of mosquitoes were also recorded. In Olama, mosquitoes are now found to bite predominantly outdoors while this was not the case previously [51]. In Nyabessan, where mosquitoes were recorded biting outdoors, the pattern was maintained despite the predominance of *An. gambiae* over local species. This behaviour could likely have resulted from the use of treated nets which are known to induce exophily in mosquito populations [21, 22]. In accordance with these findings, few mosquitoes were recorded resting indoors. Yet, a high human blood index was recorded in mosquito resting indoors and this supports a high preference for human blood. This could rather be the result of the scarcity of alternative hosts such as cattle in the area. The latter has already been emphasised in previous studies [51, 52].

Considering the night-biting cycle of mosquitoes, it appeared that mosquitoes were either feeding predominantly during the first part of the night (for species such as *An. paludis* and *An. marshallii*) or evenly during the night (*An. moucheti*) and this trend was different from previous observations [51]. The inability to detect a clear trend could result from the low sample size recorded for some species. Yet, changes in mosquito biting behaviour have been reported in previous studies and these could have resulted from a shift in mosquito composition due to the progressive replacement of more vulnerable taxa or the expression of pre-existing plastic behavioural phenotypes in response to modified resource availability [22, 53]. As such, this still deserves further investigation. Additionally, during the last two decades, an increase in temperature of 0.4 °C compared to the period 1961–1990 and a reduction in rainfall ranging from 10 to 20%, have been reported in Cameroon [54]. It is possible that these changes in weather patterns could be affecting anopheline distribution and dynamics and probably need further investigation.

Bioassays conducted with *An*. *gambiae* females indicated an increased prevalence of pyrethroid resistance in the two sites. In the absence of intensive agriculture in the area, it is likely that the use of treated nets by the population might have induced a high selective pressure on local mosquito species responsible for the emergence of insecticide resistance. The level of resistance recorded during this study was similar to insecticide resistance levels recorded across the country [2] and stresses the need for regular monitoring of local vector populations.

A decrease in malaria transmission intensity was recorded in both Olama and Nyabessan, yet the level of reduction recorded in Nyabessan was lower than that seen in Olama. It is likely that the usage of treated nets by the population might have contributed to the reduction of malaria transmission in Olama but that their impact in Nyabessan was counteracted by the construction of a dam that enabled a more efficient vector [*An. gambiae* (*s.l*.)] to invade and thus cause high transmission levels to be maintained. Since the dam construction could contribute to increasing malaria transmission risk, such projects should incorporate heightened malaria control efforts to improve population protection and reduce disease transmission. Parasitological indices indicated a high prevalence of malaria in the local human population and the variation across age classes could likely point to an uneven distribution or uptake of vector control measures as reported elsewhere [49, 55]. Children of 0 to 5 years and 5 to 16 years were found to have the highest parasite infection prevalence and this may reflect low usage of nets by this group. In addition, the construction of a dam in Nyabessan and the movement of migrants to this area seeking jobs could be an important source for infection importation from affected zones. Similar situations have been reported for Bioko island [49]. All these impediments highlight the need for improved malaria control strategies through improved detection of cases, treatment and malaria awareness campaigns in order to reduce the burden of the disease. Comparing malaria prevalence between users and non-users of treated nets, it appeared that non-users of treated nets were twice as likely to be infected by *Plasmodium falciparum* than users. This highlights the importance of treated net use despite an increased prevalence of insecticide resistance in vector populations which has to be countered in order to improve the control of malaria.

## Conclusions

This study clearly demonstrates the need for frequent monitoring and evaluation of vector control measures in order to improve malaria control strategies. Although a decrease in malaria transmission intensity and malaria prevalence have been reported both in this study and in studies conducted across the country, this level is still far from the target of the national malaria control programme. As the present study indicates, several factors including insecticide resistance, changes in vector bionomics, population non-adherence to control measures, environmental modifications such as deforestation and construction of dams, affect the effectiveness of vector control measures in the forest region and could have effects on local malaria transmission. Further study is needed in order to prioritize strategies to be implemented in the field to reduce the burden of malaria. In Cameroon, LLINs are the only tools used for vector control. With the rapid expansion of insecticide resistance, it is anticipated that these tools will soon be inefficient if strategies to mitigate its impact are not implemented. In this context, the promotion of an integrated vector control approach is recommended by the World Health Organization, and could help preserve the efficacy of existing tools and improve the control of malaria and other vector borne diseases highly prevalent in Cameroon.

## Abbreviations

CSP: Circum-sporozoite protein
EIR: Entomological inoculation rate
ELISA: Enzyme linked immunosorbent assays
GLMM: Generalized linear mixed model
HBR: Human biting rate
IRS: Indoor residual spraying
ITN: Insecticide treated nets
LLINs: long-lasting insecticidal nets
mRDTs: Malaria rapid diagnostic tests
WHO: World Health Organization
